# Non-ablative fractional 1540-nm Er:glass laser for the treatment of striae gravidarum: a case series

**DOI:** 10.3389/fmed.2026.1902791

**Published:** 2026-08-11

**Authors:** Si-Ning Wang, Lixian Xu, Mingyue Qiang, Yujuan Zhao, Wei Cao, Rui-Li Zhang

**Affiliations:** Department of Dermatology, The Second Affiliated Hospital of Nanjing Medical University, Nanjing, China

**Keywords:** striae gravidarum, striae distensae, non-ablative fractional laser, 1540-nm Er:glass laser, postpartum

## Abstract

**Background and objectives:**

Striae gravidarum (SG) is a form of striae distensae for which no single modality reliably and completely corrects the lesions, and high-level evidence remains limited. This study evaluated the clinical outcomes and safety of the 1540-nm non-ablative fractional Er:glass laser (NAFL) for SG and aimed to add real-world data in Chinese (Fitzpatrick III–IV) women.

**Patients and methods:**

We retrospectively reviewed the first cohort of consecutive postpartum women with abdominal SG who began 1540-nm NAFL treatment in our department on 31 March 2022. Efficacy was independently graded by three dermatologists who were blinded to treatment details, using a quartile improvement scale (0–3) and the 5-point Global Aesthetic Improvement Scale (GAIS); the three ratings were averaged, and inter-rater agreement was assessed with the intraclass correlation coefficient (ICC). Patient-reported satisfaction (5-point unipolar scale), pain, and adverse events were also recorded.

**Results:**

Eight patients were analyzed. The mean quartile improvement score was 1.79 ± 0.78 (corresponding to mild-to-moderate improvement) and the mean GAIS was 2.29 ± 0.88 (between “improved” and “much improved”). Inter-rater agreement was good to excellent (ICC = 0.82 and 0.93, respectively). All patients reported satisfaction with the outcome. Adverse events were limited to tolerable intraprocedural pain, transient erythema, subcutaneous petechiae (1 patient), and post-inflammatory hyperpigmentation (3 patients), all of which resolved.

**Conclusion:**

In this small, uncontrolled series, the 1540-nm NAFL appeared to be well tolerated and may provide cosmetic improvement in SG. These preliminary observations require confirmation in larger, controlled studies.

## Introduction

Striae gravidarum (SG) is a form of striae distensae, which arises due to the stretching of the dermis (1). SG develops particularly during pregnancy. There are two main types of SG: striae rubrae and striae albae. Striae rubrae are characterized by a pinkish-red or dark-purple color, whereas striae albae appear white, atrophic, and scar-like. Several modalities have been used to treat SG, including fractional lasers, radiofrequency, microneedling, and platelet-rich plasma-based combinations (2, 3). However, no single modality reliably and completely corrects striae; responses are often incomplete and variable, particularly for mature striae albae and in darker skin types, and high-level evidence remains limited.

Preclinical work suggested that the erbium glass laser can modulate metalloproteinase and interleukin expression, which may help rejuvenate and remodel the skin (4). The 1540-nm non-ablative fractional Er:glass laser (NAFL) adopts a non-ablative mode of tissue coagulation, with the stratum corneum remaining intact and tissue not being vaporized; it creates multiple microscopic thermal zones (MTZ) reaching up to 1000 μm deep surrounded by islands of viable tissue, and resurfaces with extrusion and replacement of damaged tissue, with re-epithelialization within 24 h. The 1540-nm NAFL has been used successfully to treat various types of scars. For example, Taudorf et al. (5) reported that 1540-nm NAFL induced long-term clinical and histological improvement of mature burn scars. In addition, 1540-nm NAFL was reported to be an effective treatment for acne scars, with notable improvement even in active and severe acne, and was recommended as a valuable option for safe, nonsurgical improvement in moderate or severe acne scars (6, 7). Furthermore, 1540-nm NAFL was applied to minimize scar formation during surgical wound healing (8). Given its dermal remodeling effects and clinical utility in scar management, 1540-nm NAFL may have therapeutic potential for SG.

Although 1540-nm NAFL has been investigated for the treatment of striae distensae, real-world evidence focusing on postpartum abdominal striae gravidarum in Chinese women with Fitzpatrick skin types III–IV remains limited. Therefore, this retrospective case series aimed to describe the clinical response and safety profile of 1540-nm NAFL performed with the Cynosure XD Microlens handpiece in this underrepresented population.

## Materials and methods

In this study, we retrospectively reviewed the clinical photographs of the first cohort of consecutive patients with SG who began 1540-nm NAFL treatment in our department on 31 March 2022, the date on which this modality was first introduced for SG at our institution. On that day, 13 consecutive patients started treatment; 5 were subsequently excluded (2 had been treated for striae within the previous 6 months, 1 was lactating, 1 had a history of keloids, and 1 was lost to follow-up with incomplete photographic records), leaving 8 patients for analysis. Thus, these 8 patients represent the first consecutive SG patients treated with 1540-nm NAFL at our institution. All included patients met the diagnostic criteria of abdominal SG and had an SG duration of more than 6 months postpartum. Patients were excluded if they met any of the following criteria: (1) pregnancy or lactation; (2) a history of hypertrophic scars or keloids; (3) use of photosensitizing drugs; (4) treatment for striae within the previous 6 months; or (5) incomplete records or loss to follow-up.

All eight patients were postpartum women (age range, 29–44 years; Fitzpatrick skin types III–IV) presenting with abdominal striae gravidarum of 9 months to 19 years’ duration. Fitzpatrick skin type was assessed clinically by the treating dermatologist based on baseline skin color and patient-reported sun reactivity, including the tendency to burn or tan after sun exposure. Their main concern was the cosmetic appearance of the abdominal striae, without associated pain or functional impairment. None of the included patients had received treatment for striae within the previous 6 months, and none were pregnant or lactating at the time of treatment. None had a personal or family history of hypertrophic scars or keloids, connective-tissue disorders, or other relevant genetic conditions, and none were receiving systemic corticosteroids or photosensitizing medications.

Treatment was performed with a 1540-nm non-ablative fractional Er:glass laser (Icon platform, Cynosure) using the XD Microlens handpiece, which delivers a 12 mm × 12 mm spot (∼25 microbeams/cm^2^) designed for deeper dermal penetration suited to scars and striae. A pulse duration of 15 ms was used, with built-in advanced contact cooling (contact window maintained at ∼5 °C) applied throughout to protect the epidermis. Topical anesthesia with compound lidocaine cream (lidocaine 2.5%/prilocaine 2.5%) was applied under occlusion for 30 min before each session. Each region received 3 sequential passes per session at fluences of 50–70 J/cm^2^, individualized to lesion type, skin response, and tolerance (Table 1). The clinical treatment endpoint was mild erythema with perifollicular edema. Sessions were repeated at intervals of at least 4 weeks, and patients received 1–5 sessions in total (Table 1). After each session, cold compresses were applied for 30 min, and patients were advised to use bland emollients and broad-spectrum sunscreen and to avoid sun exposure.

**TABLE 1:** Clinical characteristics and treatment information of the patients.

Case number	1	2	3	4	5	6	7	8
Fitzpatrick skin type	III	IV	III	III	III	III	III	III
Age, years	36	44	30	41	41	29	40	34
History of SG	10 years	19 years	3 years	9 years	12 years	9 months	12 years	6 years
Type of SG	Striae albae	Striae albae	Striae albae	Striae albae	Striae albae	Striae rubrae	Striae albae	Striae albae
Anatomic location	Both sides of the abdomen and around the umbilicus	Lower abdomen	Around the umbilicus and both sides of the lower abdomen	Middle and lower abdomen	Whole abdomen	Both sides of the abdomen	Both sides of the abdomen and around the umbilicus	Whole abdomen
No. of NAFL treatments	1	2	2	3	3	4	5	5
Fluence settings (J/cm^2^; 3 passes per region per session)	50-55-60	50∼55-55∼60-65	50∼60-55∼65-60∼65	50-55∼60-60∼65	50∼65-55∼65-60∼70	50∼55-55∼60-60∼65	50∼60-55∼60-65	50∼60-55∼65-60∼65
Pain during treatment	Tolerated	Tolerated	Tolerated	Tolerated	Tolerated	Tolerated	Tolerated	Tolerated
Short-term erythema	Y	Y	Y	Y	Y	Y	Y	Y
PIH after treatment	N	Y	Y	N	N	N	Y	N
Subcutaneous petechiae	N	Y	N	N	N	N	N	N
Persistent adverse events during available follow-up	N	N	N	N	N	N	N	N
Quartile improvement scale	1.67 ± 0.58	2.33 ± 0.58	2.00 ± 0.00	0.67 ± 0.58	1.33 ± 1.15	3.00 ± 0.00	2.33 ± 0.58	1.00 ± 0.00
GAIS	2.00 ± 0.00	3.00 ± 0.00	2.33 ± 0.58	1.33 ± 0.58	1.33 ± 0.58	4.00 ± 0.00	2.33 ± 0.58	2.00 ± 0.00
Patient’s treatment satisfaction	Very satisfied	Very satisfied	Very satisfied	Moderately satisfied	Very satisfied	Completely satisfied	Very satisfied	Very satisfied
Visual follow-up after last treatment	Approximately 1 month (all patients)							

SG, striae gravidarum; NAFL, non-ablative fractional laser; PIH, post-inflammatory hyperpigmentation; GAIS, Global Aesthetic Improvement Scale; Y, yes; N, no. Within each treatment session, each region received 3 passes; values separated by hyphens denote the fluences used for the three sequential passes, and values connected by a tilde denote the range of fluences used for that pass across different sessions. Fluences were titrated across sessions within these ranges. Quartile improvement scale: 0 = no improvement/worsening (<25%), 1 = mild (25%–50%), 2 = moderate (51%–75%), 3 = marked (76%–100%). GAIS: 0 = worse, 1 = no change, 2 = improved, 3 = much improved, 4 = very much improved. Quartile and GAIS values are presented as mean ± SD of three independent, blinded raters. Visual efficacy follow-up was performed approximately 1 month after the final treatment; adverse events were followed by telephone until resolution when applicable.

The diagnosis of striae gravidarum was made clinically, based on the characteristic morphology and distribution of the lesions: linear atrophic bands lying perpendicular to the lines of skin tension over the abdomen, arising in the postpartum period. Lesions were classified as striae rubrae (erythematous to violaceous, early) or striae albae (hypopigmented, atrophic, mature). The differential diagnosis of linear atrophic skin lesions includes linear focal elastosis, anetoderma and atrophic scarring; these were considered unlikely given the typical postpartum onset, distribution and appearance, and no patient had features to suggest them, so skin biopsy and laboratory testing were not required. Because the diagnosis and the assessment of severity rely on visual features, standardized photography was used to document baseline area, color, width and degree of atrophy and to compare with post-treatment images; the inherent variability of photographic evaluation (lighting, angle, positioning) was minimized by standardizing the setup. In terms of prognosis, striae rubrae may fade partially over time, whereas striae albae are mature lesions that tend to persist and are generally more resistant to treatment.

The primary efficacy outcome was the improvement in the overall appearance of SG, assessed by comparing standardized pre- and post-treatment photographs (digital camera; Sony, Tokyo, Japan). Clinical efficacy was independently assessed by three dermatologists who had not participated in the treatments and were blinded to patient identifiers, treatment parameters, and patient-reported satisfaction. For each patient, baseline and post-treatment photographs were compared and graded using a quartile improvement scale (0 = no improvement/worsening [<25%]; 1 = mild [25%–50%]; 2 = moderate [51%–75%]; 3 = marked [76%–100%] improvement) and the 5-point Global Aesthetic Improvement Scale (GAIS: 0 = worse, 1 = no change, 2 = improved, 3 = much improved, 4 = very much improved) (9, 10). Each rater scored the photographs independently and was blinded to the other raters’ assessments. The three scores were averaged for each patient. Inter-rater agreement was assessed using the intraclass correlation coefficient (ICC; two-way random-effects, absolute agreement, average measures). Patient-reported satisfaction was assessed separately using a 5-point unipolar satisfaction scale (not at all, slightly, moderately, very, and completely satisfied). The incidence and severity of adverse events were recorded to evaluate safety. Continuous data are presented as mean ± standard deviation (SD). Photographs for efficacy assessment were obtained at each visit, approximately 4 weeks after the preceding treatment session; the final efficacy evaluation was therefore based on images obtained approximately 1 month after the last treatment. Patients who developed post-inflammatory hyperpigmentation (PIH) or other adverse events received additional telephone follow-up until the events resolved; in these patients, PIH had resolved by 3 months. No further long-term follow-up beyond this period was performed.

## Case description

**FIGURE 1:**
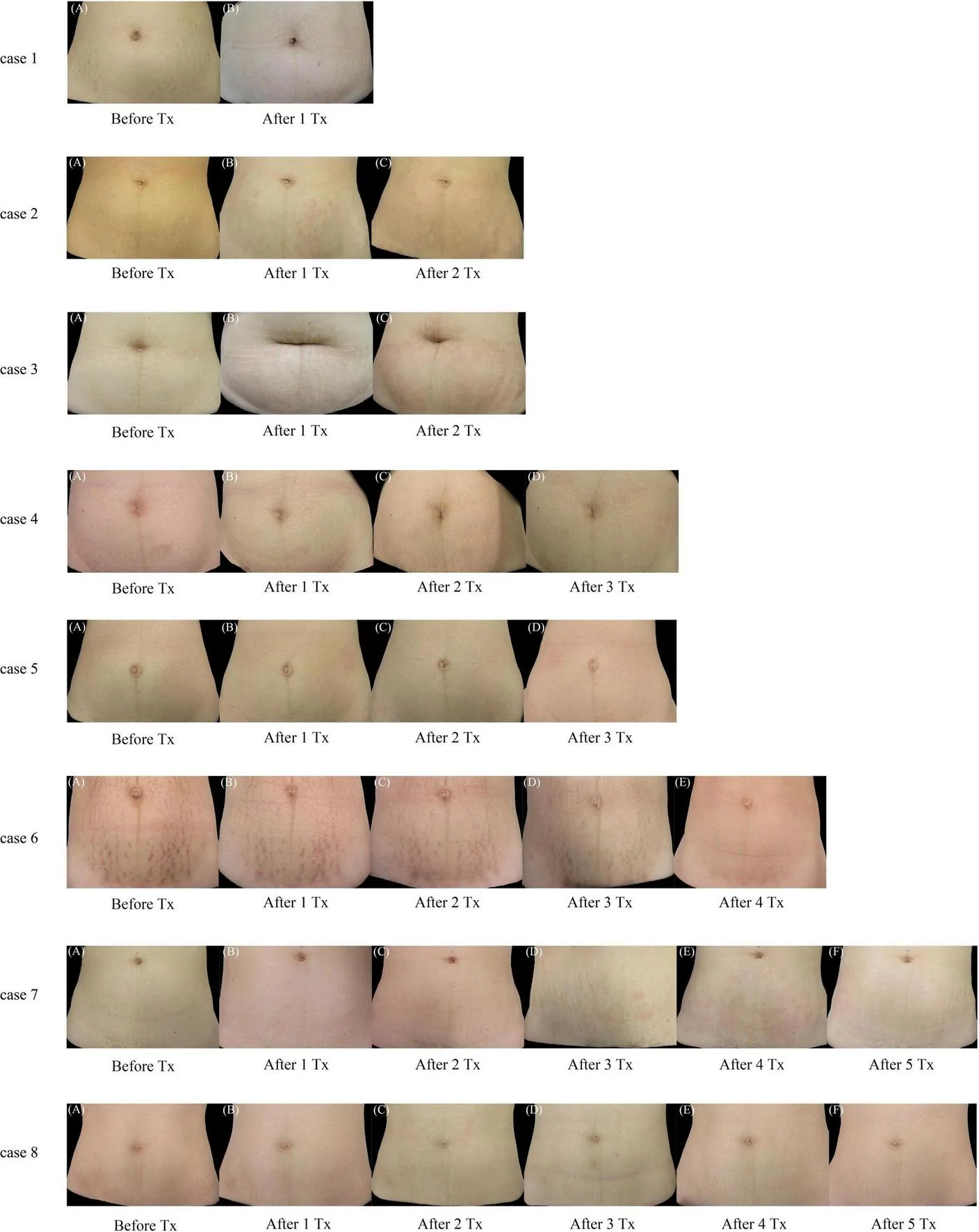
Clinical photographs of the eight patients before and after treatment. Case 1 (B): after 1 treatment, the overall area of SG appeared markedly decreased. Case 2 (C): after 2 treatments, striae albae were less atrophic and more even in color, but the treated area presented slight PIH. Case 3 (C): after 2 treatments, most of the striae on the lower abdomen were flattened and became closer to the surrounding normal skin color, but the laser-treated area displayed a PIH pattern. Case 4 (D): after 3 treatments, striae albae became flatter and thinner in width without PIH, especially on the left side of the abdomen. Case 5 (D): after 3 treatments, striae albae were decreased in area, faded in color, flattened, and narrowed in width without PIH. Case 6 (E): after 4 treatments, striae rubrae were largely resolved without PIH, leaving only some thin striae albae. Case 7 (F): after 5 treatments, most striae albae faded with slight PIH. Case 8 (F): after 5 treatments, the width and area of striae albae, especially on the lower abdomen, decreased without PIH. Tx, treatment; PIH, post-inflammatory hyperpigmentation; SG, striae gravidarum.

De-identified clinical and treatment details, together with the individual quartile and GAIS scores for each patient, are summarized in Table 1, and representative pre- and post-treatment photographs are shown in Figure 1.

### Case 1

A 36-year-old woman with 10 years of SG presented with striae albae on both sides of the abdomen and around the umbilicus (Figure 1, 1A). She received one treatment with 1540-nm NAFL (3 passes per region, using 50–60 J/cm^2^). After one treatment, the overall area of SG appeared markedly decreased. The width of striae albae was markedly reduced and the color of striae albae was improved. Most striae albae were less atrophic and flatter without post-inflammatory hyperpigmentation (PIH). In general, the patient’s skin appeared uniformly smooth (Figure 1, 1B). This patient reported being very satisfied with the treatment result.

### Case 2

A 44-year-old woman with 19 years of SG presented with several striae albae on the lower abdomen (Figure 1, 2A). She received two treatments with 1540-nm NAFL (3 passes per region, using 50–65 J/cm^2^). Before the second treatment, subcutaneous petechiae were observed in the treated area (Figure 1, 2B). After two treatments, the number of striae albae was decreased, but the treated area presented slight PIH. Although the width of striae albae was not changed substantially, striae albae were less atrophic and more even in color (Figure 1, 2C). At 3 months of follow-up by phone call, the patient reported that the PIH had resolved and that she was very satisfied with the treatment result.

### Case 3

A 30-year-old woman with 3 years of SG presented with striae albae on both sides of the lower abdomen and around the umbilicus (Figure 1, 3A). She received two treatments with NAFL (3 passes per region, using 50–65 J/cm^2^). Before the second treatment, the color of the striae on the lower abdomen became lighter. The therapeutic effect on striae albae around the umbilicus was less obvious, possibly because of weight gain (Figure 1, 3B). After two treatments, the width of striae was narrower and the color of striae albae was faded on the lower abdomen and around the umbilicus. Most of the striae on the lower abdomen were flattened and became closer to the surrounding normal skin color. The overall number of striae was markedly reduced. However, the laser-treated area displayed a PIH pattern (Figure 1, 3C). At 3 months of phone-call follow-up, the patient reported that the PIH had resolved. This patient reported being very satisfied with the treatment outcome.

### Case 4

A 41-year-old woman with 9 years of SG presented with striae albae on the middle and lower abdomen (Figure 1, 4A). She received three treatments with NAFL (3 passes per region, using 50–65 J/cm^2^). Before the second treatment, part of the striae albae on the left side of the abdomen became flatter (Figure 1, 4B). Before the third treatment, the color of striae, especially around the umbilicus, became lighter (Figure 1, 4C). After the third treatment, striae albae became flatter and thinner in width without PIH, especially on the left side of the abdomen. The total area of striae albae appeared smaller and the patient showed modest changes in color and texture on non-blinded clinical review (Figure 1, 4D). This patient reported being moderately satisfied with the result of the treatment.

### Case 5

A 41-year-old woman with 12 years of SG presented with striae albae on the whole abdomen (Figure 1, 5A). She received three treatments with NAFL (3 passes per region, using 50–70 J/cm^2^). Before the second treatment, striae albae on both sides of the abdomen were faded in color (Figure 1, 5B). Before the third treatment, most striae albae became flatter and were decreased in number (Figure 1, 5C). After the third treatment, striae albae were decreased in area, faded in color, flattened, and narrowed in width without PIH (Figure 1, 5D). This patient reported being very satisfied with the treatment result.

### Case 6

A 29-year-old woman with 9 months of SG presented with numerous striae rubrae on both sides of the abdomen (Figure 1, 6A). She received four treatments with NAFL (3 passes per region, 50–65 J/cm^2^). Before the second treatment, a few striae rubrae became lighter in color (Figure 1, 6B). Before the third treatment, the atrophy of striae rubrae became flatter (Figure 1, 6C). Before the fourth treatment, the area and width of striae rubrae were apparently reduced (Figure 1, 6D). After the fourth treatment, the striae rubrae were largely resolved without PIH, leaving only some thin striae albae (Figure 1, 6E). This patient reported being completely satisfied with the result of the treatment.

### Case 7

A 40-year-old woman with 12 years of SG presented with several striae albae on both sides of the abdomen and around the umbilicus (Figure 1, 7A). She received five treatments with NAFL (3 passes per region, using 50–65 J/cm^2^). Before the second treatment, the color of several striae albae became lighter (Figure 1, 7B). Before the third treatment, striae albae on the lower left abdomen became flatter (Figure 1, 7C). Before her fourth treatment, striae albae on the lower right abdomen were still obvious. We then performed energy-increasing treatment on the striae albae on the lower right abdomen (Figure 1, 7D). Before the fifth treatment, the width of striae albae on the lower left abdomen was apparently narrowed, with PIH. At the same time, striae albae around the umbilicus were virtually invisible. The area of striae albae was decreased overall (Figure 1, 7E). After the fifth treatment, most striae albae faded with slight PIH (Figure 1, 7F). At 3 months of phone-call follow-up, she reported that PIH had disappeared. This patient reported being very satisfied with the treatment results.

### Case 8

A 34-year-old woman with 6 years of SG presented with striae albae on the whole abdomen (Figure 1, 8A). She received five treatments with NAFL (3 passes per region, using 50–65 J/cm^2^). Before the second treatment, several striae albae became thinner in width (Figure 1, 8B). On the day before the third treatment, striae albae on the lower left abdomen became flatter (Figure 1, 8C). Before the fourth treatment, striae albae on the lower right abdomen and around the navel also became flatter and decreased in area (Figure 1, 8D). Before the fifth treatment, the overall color of striae albae became lighter (Figure 1, 8E). After the fifth treatment, the width and area of striae albae, especially on the lower abdomen, decreased without PIH. The patient demonstrated visible improvement in both atrophy and color (Figure 1, 8F). This patient reported being very satisfied with the treatment outcome.

## Outcomes and follow-up

In this study, the duration of SG of the 8 patients ranged from 9 months to 19 years. These patients received a total of 25 NAFL treatments. Among them, 3 patients (37.5%) had ≥4 treatments, 2 patients (25%) received 3 treatments, 2 patients (25%) received 2 treatments, and 1 patient (12.5%) received 1 treatment. The time interval between laser sessions was at least 4 weeks. All patients showed some degree of clinical improvement and were satisfied with treatment. Across the eight patients, the mean quartile score was 1.79 ± 0.78 (mild-to-moderate improvement) and the mean GAIS was 2.29 ± 0.88 (between “improved” and “much improved”) (Table 1). Inter-rater agreement among the three blinded dermatologists was good for the quartile scale (ICC = 0.82; 95% CI 0.43–0.96) and excellent for GAIS (ICC = 0.93; 95% CI 0.77–0.98). All eight patients also reported satisfaction with the treatment outcome: six (75.0%) were very satisfied, one (12.5%) completely satisfied, and one (12.5%) moderately satisfied. All eight patients reported that pain during treatment was tolerable and resolved within a few hours. After treatment, short-term erythema, as expected, was seen immediately in all 8 patients, which typically resolved within 2–7 days. One patient developed subcutaneous petechiae hours after the end of treatment. PIH developed in three patients during or shortly after the treatment course, all resolving within 3 months. No persistent adverse events or worsening of SG were observed during the available follow-up.

## Discussion

The treatment of SG remains challenging. In recent years, laser therapy has become an important treatment option for SG. Compared with ablative lasers, 1540-nm NAFL may cause less epidermal disruption and shorter downtime, and has been used in scar modulation with acceptable tolerability (8, 11).

Histologically, striae rubrae represent an early, inflammatory stage (dermal edema, perivascular lymphocytic infiltrate), whereas striae albae are mature, atrophic-scar-like lesions (epidermal atrophy, loss of rete ridges, reduced vascularity, fragmented elastic fibers, and densely packed horizontal collagen bundles). The 1540-nm Er:glass NAFL delivers columnar microthermal zones into the dermis while sparing the epidermis, triggering a wound-healing response that increases epidermal and dermal thickness and promotes collagen I/III remodeling and elastin regeneration (9, 12). Because earlier rubrae lesions retain greater vascularity and fibroblast activity, they may be more responsive than mature albae lesions; nevertheless, dermal remodeling induced by 1540-nm NAFL provides a rationale for its use in chronic (>6 months) postpartum abdominal striae, including the more treatment-resistant striae albae.

Several studies support the use of the 1540-nm Er:glass NAFL for striae. de Angelis et al. reported safe and efficacious results in Fitzpatrick skin types II–IV, with blinded evaluators reporting an overall mean improvement of 51%–75% on photographs taken at least 3 months after treatment (9). Alves et al. successfully treated corticosteroid-induced striae distensae with the 1540-nm NAFL in Fitzpatrick phototype IV patients (13), and Malekzad et al. reported improvement of striae alba with the 1540-nm XD probe in Fitzpatrick III–V skin (14). In Asian patients, Yang and Lee found non-ablative fractional Er:glass and ablative CO_2_ lasers to be comparably effective for abdominal striae (15). A further comparative study showed that both the 1540-nm and 1410-nm NAFL improved striae distensae clinically and histopathologically (12). Our findings are consistent with this literature and extend it to postpartum SG in Chinese (Fitzpatrick III–IV) women.

Beyond energy-based devices, regenerative approaches are emerging for striae distensae, particularly for treatment-resistant striae albae. Autologous platelet-rich plasma (PRP) and nanofat–rich in growth factors and adipose-derived stromal/stem cells–can stimulate fibroblast activity and collagen synthesis. In a randomized study of abdominal striae albae, both PRP and nanofat significantly increased type I collagen production compared with controls, and their combination produced a synergistic effect with superior clinical improvement (16). Because the non-ablative fractional laser remodels the dermis through a controlled wound-healing response, whereas these regenerative therapies supply growth factors and progenitor cells, the two mechanisms are potentially complementary; combining NAFL with PRP or nanofat may further enhance outcomes–especially for mature striae albae–and warrants investigation in future controlled studies.

Efficacy outcomes reflect the appearance approximately 1 month after the last treatment session. In this series, 1540-nm NAFL produced mild-to-moderate improvement on the quartile scale and improvement-to-much-improvement on GAIS, as assessed by blinded evaluators, with good-to-excellent inter-rater agreement (quartile ICC = 0.82; GAIS ICC = 0.93), and all patients were satisfied with the outcome. Clinically, the laser not only reduced the width and area of striae but also improved their color and atrophy. The number of sessions and the stepwise energy escalation were individualized: more extensive or treatment-resistant lesions (typically wider or more atrophic striae albae) received additional sessions and higher fluences when the response to prior sessions was incomplete and no significant adverse reaction had occurred. The single patient with striae rubrae (Case 6) showed the highest improvement scores, consistent with prior reports that earlier lesions may respond more readily than mature striae albae. However, with only one rubrae case and no quantitative subgroup analysis, no comparative conclusion can be drawn. Adverse events were limited and transient: erythema appeared immediately and resolved within 2–7 days, subcutaneous petechiae resolved within hours to days, and PIH (3 patients) resolved within 3 months.

The strengths of this series include the use of two standardized improvement scales scored independently by three blinded, non-treating dermatologists with good-to-excellent inter-rater agreement, which reduces observer bias, and a focus on an underrepresented population (postpartum Chinese women with Fitzpatrick III–IV skin). The magnitude of improvement we observed is broadly consistent with previous reports of 1540-nm NAFL for striae, which supports the biological plausibility of a treatment effect. Nevertheless, in the absence of a control group, we cannot exclude contributions from natural maturation of the striae, regression to the mean, or concurrent skin care, and causality should not be inferred from this uncontrolled design. Several limitations should be considered when interpreting these findings. First, the sample size is small, and the design is retrospective and uncontrolled, so the efficacy observations are preliminary and not generalizable. Second, no histological assessment was performed, and efficacy was based on photographic grading and patient-reported satisfaction rather than objective instrumental measurements. Third, the number of sessions and treatment intervals varied between patients, which may have influenced the outcomes. Fourth, follow-up was limited: efficacy was assessed at approximately 1 month after the final treatment, and only patients with adverse events underwent further (telephone) follow-up. The durability of improvement beyond 1 month was not systematically evaluated, and longer prospective follow-up is needed. Finally, photographic standardization is inherently difficult for striae because of variation in lighting, angle, and patient positioning. Given the small, uncontrolled, retrospective design and short follow-up, these findings should be regarded as preliminary, hypothesis-generating observations rather than confirmatory evidence of efficacy. Larger, prospective, controlled studies with standardized parameters, objective measurements, and longer follow-up are needed to confirm these findings.

## Conclusion

In this small, uncontrolled case series, 1540-nm NAFL appeared to be well tolerated and may provide cosmetic improvement in patients with SG. However, given the retrospective design, small sample size, lack of a control group, absence of objective measurements, and limited follow-up, these preliminary findings require confirmation in larger prospective controlled studies with standardized treatment parameters and longer follow-up.

## Data Availability

The raw data supporting the conclusions of this article will be made available by the authors, without undue reservation.
